# Effects of Hot-Air Drying Temperature on Biochemical Quality, Drying Kinetics, and Predictive Modeling of *Ziziphus jujuba* Fruits

**DOI:** 10.3390/molecules31162903

**Published:** 2026-08-20

**Authors:** Hakan Keles, Adil Koray Yildiz, Burcu Aksüt, Nalan Bakoğlu, Müjgan Güney, Nesibe Ebru Kafkas

**Affiliations:** 1 Department of Horticulture, Faculty of Agriculture, University of Yozgat Bozok, Yozgat 66100, Türkiye; mujgan.guney@yobu.edu.tr; 2 Department of Agricultural Machinery and Technologies Engineering, Faculty of Agriculture, University of Yozgat Bozok, Yozgat 66100, Türkiye; adilkorayyildiz@gmail.com; 3 Department of Biosystem Engineering, Faculty of Agriculture, University of Tokat Gaziosmanpaşa, Tokat 60250, Türkiye; burcu.aksutt@gmail.com; 4 Department of Horticulture, Faculty of Agriculture, University of Recep Tayyip Erdoğan, Pazar Rize 53300, Türkiye; nalan.bakoglu@erdogan.edu.tr; 5 Department of Horticulture, Faculty of Agriculture, University of Cukurova, Adana 01330, Türkiye; ebruyasakafkas@gmail.com

**Keywords:** jujube, hot-air drying, drying kinetics, artificial neural network, phenolic compounds (SDG12), antioxidant activity (SDG3)

## Abstract

Jujube (*Ziziphus jujuba* Mill.) is an important functional fruit with high levels of bioactive compounds. However, its high moisture content limits its postharvest shelf life. The biochemical properties of jujube fruits influenced by hot-air drying at 50, 60, and 70 °C were studied and predictive models were evaluated to optimize drying. The drying kinetics were modeled using the six classical thin-layer models and artificial neural network (ANN) approach. The Midilli–Kucuk model was the best classical model, and the ANN model showed the best predictive performance, with the RMSE values improved up to 19.52% in the independent validation dataset. The drying temperature significantly influenced the color, composition of sugars, organic acids, total phenolic content, and antioxidant activity. Color difference (ΔE = 5.88) and browning index of the 70 °C treatment showed less color change, indicating better color retention. Sugar and organic acid concentrations were significantly higher in dried fruits than in fresh fruits. The highest total phenolic content (5393.43 mg GAE 100 g^−1^) and DPPH radical scavenging activity (73.56%) were found at 50 °C and progressively decreased with increasing drying temperature. Multivariate analyses further showed clear biochemical differentiation between fresh and dried samples according to drying temperature. Generally, the drying temperature had a significant effect on the nutritional and functional quality of jujube fruits. Although 50 °C was the most effective for maintaining phenolic compounds and antioxidant activity, 70 °C offered better color retention and shorter drying time. The ANN method was a useful tool to predict the drying behavior and optimize processing conditions in the production of jujube fruit.

## 1. Introduction

Plant biodiversity constitutes a fundamental resource for the sustainability of natural ecosystems and provides an essential genetic reservoir for crop improvement and the development of functional foods with health-promoting properties [1]. In this context, resilient plant species adapted to harsh environmental conditions have gained increasing attention due to their potential contributions to sustainable agriculture and food security.

Among these plants, jujube is a valuable resource due to its exceptional tolerance to drought, heat, and salinity; this characteristic makes it possible to be cultivated in arid and semi-arid regions where conventional crops often fail. The genus *Ziziphus* includes several species with economic and nutritional importance, such as *Ziziphus jujuba* Mill., native to northern China [2], and *Ziziphus mauritiana* Lam. (Indian jujube), which originates from India and Pakistan [3,4]. In addition to these primary centers of origin, diverse native populations and ecotypes have evolved in different geographical regions, including Iran and Türkiye, reflecting the species’ high adaptability to varying climatic conditions [1,5,6].

*Ziziphus jujuba* Mill., a member of the Rhamnaceae family within the order Rosales, has been cultivated for more than 4000–5000 years and is currently distributed across tropical and subtropical regions worldwide [7,8,9,10,11,12]. It is a semi-deciduous shrub or small tree that is well adapted to dry and harsh environmental conditions due to its high tolerance to drought, salinity, and heat [4,7]. This fruit is a drupe that undergoes distinct developmental stages characterized by color changes from green to white, yellow, and finally reddish-brown, closely related to physiological maturity and changes in biochemical composition. Fruit growth and ripening are influenced by environmental factors such as temperature, water availability, and light intensity, leading to simultaneous changes in color, texture, aroma, and flavor that determine consumer acceptance [13].

The long history of cultivation of jujube is closely linked to its nutritional value and medicinal importance, leading to its widespread use in traditional diets and therapeutic practices. The fruit is consumed in various forms, including fresh, dried, and processed products, demonstrating its versatility and economic importance within food systems [14,15].

Jujube contains phenolic compounds, soluble sugars, and organic acids that contribute to its nutritional quality and antioxidant capacity [6,8,9,10,13,16]. These compounds are associated with important biological activities and have attracted increasing interest as functional food components [2,15,17,18,19].

Despite its rich biochemical properties, preserving the quality of jujube fruit remains a significant challenge due to its high moisture content and susceptibility to rapid post-harvest deterioration. Fresh jujube fruits are susceptible to microbial spoilage and enzymatic degradation, leading to a shortened shelf life and significant post-harvest losses [3,9]. Therefore, using effective storage techniques is essential to preserve the nutritional and functional health of fruits. Among these techniques, drying is one of the most commonly used methods to extend shelf life, reduce water activity, and improve storage stability [9]. The drying process not only extends shelf life but also facilitates transportation and ensures year-round availability of jujube products, making it a vital step for food processing and industrial applications [9].

Drying is not simply the physical removal of water; it also leads to complex biochemical and physicochemical changes that can significantly affect product quality, such as color and nutritional value. Various drying techniques, including hot-air drying, infrared drying, vacuum drying, convective drying, and freeze drying, have been extensively investigated to evaluate their impact on the biochemical composition of jujube [2,9]. These methods significantly affect the composition of key bioactive compounds. Drying temperature has been shown to influence total phenolic content and antioxidant capacity, although some studies have reported that different drying methods do not significantly alter these quality attributes, suggesting that certain bioactive compounds remain relatively stable under specific processing conditions [9,20]. Although higher temperatures accelerate moisture removal and shorten drying time, they may also compromise product quality [20,21]. Therefore, optimization of drying conditions should consider not only drying efficiency but also the preservation of quality attributes.

Similarly, drying temperature has been shown to affect the degradation of water-soluble vitamins and reduce total phenolic content and antioxidant capacity [18]. However, some studies have shown that different drying methods do not significantly alter total phenolics, flavonoids, or overall antioxidant activity, suggesting that some bioactive compounds are relatively stable under certain conditions [9]. Temperature and drying time can increase drying speed but also lead to quality losses. Studies on jujube have shown that hot-air drying reduces drying time [18,19]. Therefore, not only drying time but also quality indicators should be evaluated together in determining the appropriate drying conditions.

Thin-layer drying models are widely used to explain the drying behavior of agricultural products. Models such as Lewis, Page, Henderson–Pabis, Logarithmic, Wang-Singhen, and Midilli–Kucuk are frequently preferred in describing drying kinetics in different products. However, the literature shows that no single model universally provides the best results for every product and every drying condition [20,22]. In recent years, artificial neural networks have emerged as a powerful alternative in modeling nonlinear relationships in drying processes. They are particularly successful in moisture content estimation [23].

Color measurements play an important role in evaluating the effect of the drying process on quality. The L*, a*, and b* values obtained with the CIE LAB color system, and the parameters derived from them such as chroma, hue, total color difference, and browning index, enable the objective determination of changes in product quality [24,25,26].

Previous jujube drying studies have focused on drying kinetics, moisture removal behavior, and a limited number of quality characteristics, mostly using different drying techniques such as freeze-drying, infrared drying, vacuum drying, or conventional hot-air drying. Furthermore, significant variability exists between studies due to differences in genotypes, growing environments, and post-harvest conditions [27,28,29]. To our knowledge, no previous study has simultaneously integrated thin-layer drying kinetics, ANN-based prediction, biochemical characterization (sugars, organic acids, total phenolics, antioxidant activity), and multivariate statistical analyses (PCA and heatmap) to determine the optimum hot-air drying conditions for jujube fruits grown under Turkish ecological conditions. Additionally, information on the effects of hot-air drying on the biochemical quality characteristics of jujube fruits grown under Türkiye’s ecological conditions is still insufficient. Therefore, this study comprehensively evaluates the effect of different hot-air-drying temperatures on the quality characteristics of jujube fruits grown in Türkiye, while also validating drying prediction models and determining optimum drying conditions for industrial processing and nutritional quality preservation.

Therefore, the present study aimed to comparatively characterize the biochemical properties of fresh and hot-air-dried jujube fruits, to evaluate drying dynamics using classical thin-layer models and artificial neural network approaches, and to develop a predictive modeling approach to determine optimum drying conditions. Specifically, the effects of different drying temperatures on color changes and key biochemical parameters, including total phenolic content and antioxidant capacity, were investigated.

## 2. Results and Discussion

### 2.1. Initial Moisture Content and Drying Time

The initial moisture content of jujube samples was determined to be an average of 76.54% on a wet basis. Drying continued until the samples reached a constant weight, which was used as the endpoint criterion. Drying time decreased significantly depending on temperature; total drying times were determined as 36.5, 22.5, and 13.5 h at 50, 60, and 70 °C, respectively. These results indicate that the drying rate increased as drying temperature increased.

### 2.2. Effects of Drying Temperature on Color Characteristics of Fresh and Dried Jujube Fruits

**Figure 1:**
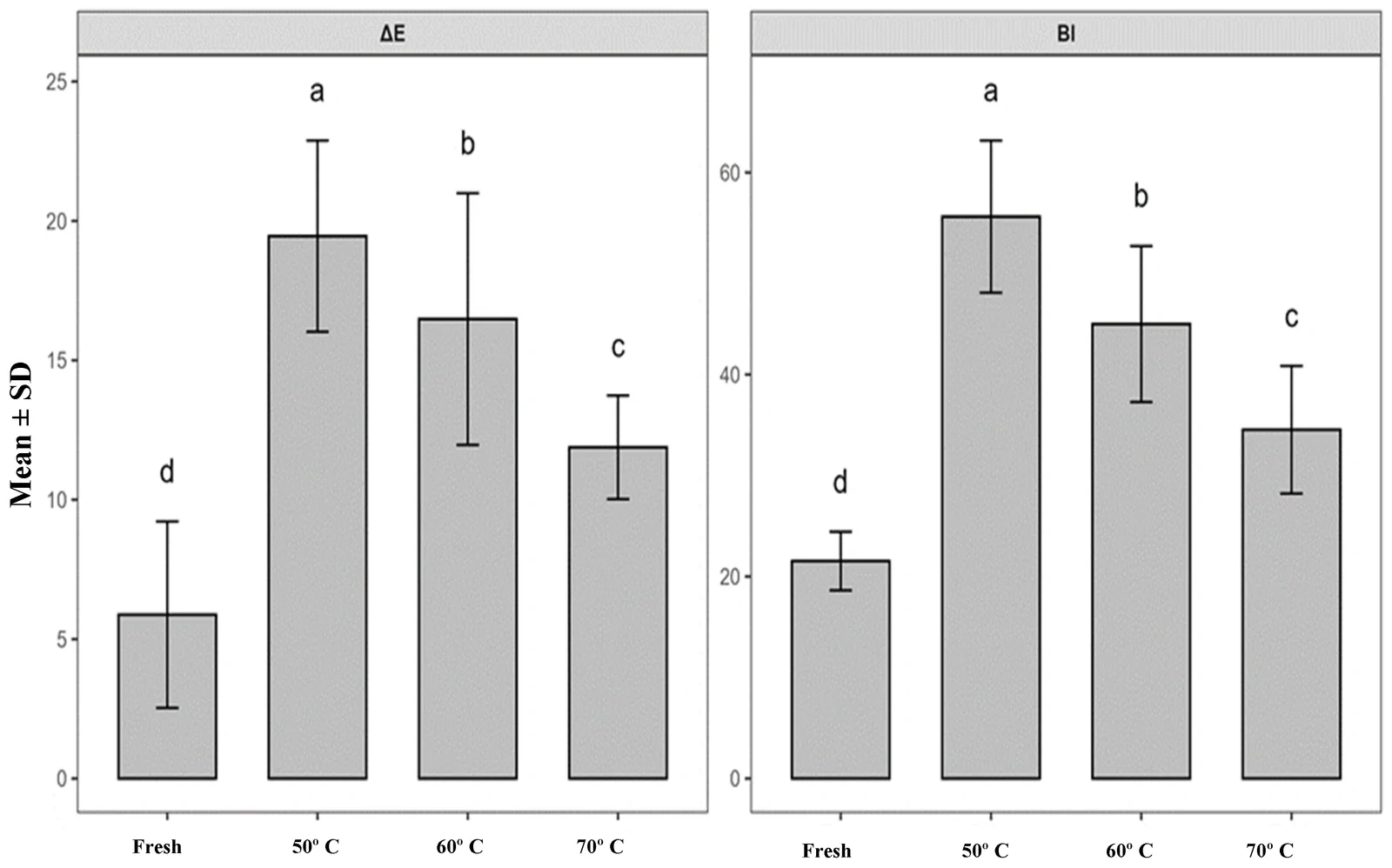
Changes in Total Color Difference (ΔE) and Browning Index (BI) of Fresh and Dried Jujube Fruits Subjected to Different Drying Temperatures.

Significant differences were determined between fresh and dried samples in terms of color parameters. Changes were observed in all L*, a*, b*, C*, h°, ΔE, and BI values as a function of drying temperature. Among the dried samples, particularly with respect to ΔE and BI values, the 70 °C treatment was found to be more favorable due to its smaller color difference and lower browning index. Details of the color data are presented in Figure 1.

Color results showed that drying temperature significantly affected jujube quality. Especially when considering the ΔE and BI values, the 70 °C application showed a more advantageous appearance among the dried samples. This suggests that drying in a shorter time may have limited the total thermal load. The literature also reports that drying causes significant changes in color parameters [19,25,26].

### 2.3. Effects of Drying Temperature on Biochemical Characteristics and Bioactive Compounds

#### 2.3.1. Effects of Drying Temperature on Sugar Composition

The sugar composition of fresh and dried jujube fruits was significantly affected by drying temperature (*p* ≤ 0.01) (Table 1). Drying increased the concentrations of all analyzed sugars compared with fresh fruits. This can be explained primarily by the large decrease in moisture content during drying, although biochemical transformations occurring during the drying process may also contribute to changes in soluble sugar composition [30].

Sucrose content ranged from 7.68% in fresh fruits to 29.32% in fruits dried at 50 °C. The highest sucrose concentration was obtained at 50 °C, followed by 60 °C (19.96%) and 70 °C (17.76%). In contrast, glucose and fructose contents reached their highest levels at 70 °C, at 24.26% and 24.62%, respectively. The lowest glucose (4.72%) and fructose (4.61%) contents were found in fresh fruit. The increase in glucose and fructose at higher drying temperatures may be due to the partial hydrolysis of sucrose into its constituent monosaccharides during heat treatment.

Among the drying processes, 70 °C resulted in the highest accumulation of reducing sugars, while 50 °C was more effective in preserving sucrose. Overall, the drying process significantly increased the sugar concentration in jujube fruits compared to fresh samples, and the amount of change varied depending on the drying temperature. The sugar concentrations determined in fresh fruits were generally within the ranges previously reported for jujube [9]. Sagbas et al. [1] reported fructose, glucose, and sucrose contents ranging from 4.86 to 13.82%, 3.35 to 9.15%, and 0.18 to 3.10%, respectively. Similarly, Keles [31] reported fructose, glucose, and sucrose concentrations of 4.2–5.1%, 2.1–2.4%, and 0.4–0.6%, respectively. The fresh fruit values obtained in the present study are therefore consistent with previously published data, although the sucrose concentration was higher than that reported by the author. Similarly, previous studies showed that total sugar content ranged between 9% and 14% in fresh jujube fruits [6,8], suggesting that jujube fruits are rich in soluble carbohydrates.

Drying markedly increased sugar concentrations as a consequence of moisture loss and water removal during the dehydration process.

The sugar profile observed in the dried samples was also comparable to the values reported for dehydrated jujube products. Yan et al. [12] reported fructose and glucose concentrations ranging from 15 to 34% and from 15 to 33%, respectively, in lyophilized fruits, which closely agrees with the fructose (20.72–24.62%) and glucose (21.55–24.26%) contents obtained in the present study. These findings indicate that, regardless of the drying method used, drying or freeze-drying concentrates the major monosaccharides to similar levels.

According to the results, sucrose reached its maximum concentration at 50 °C and gradually decreased by approximately 39.4% as the drying temperature increased to 70 °C, whereas that of glucose and fructose increased by 5.3% and 15.3%, respectively. This opposite trend is consistent with partial thermal hydrolysis (inversion) of sucrose into its constituent reducing monosaccharides during drying under high temperatures [32]. Besides moisture loss, which concentrates soluble solids, sucrose inversion increases the availability of glucose and fructose, both of which are more chemically reactive than sucrose. Because glucose and fructose are reducing sugars, they may serve as substrates for the Maillard reaction by reacting with free amino groups to form intermediate compounds and, ultimately, brown melanoidins [24,33]. However, browning development depends not only on reducing sugar concentration but also on drying temperature, processing time, amino acid availability, and water activity [24]. Although fruits dried at 70 °C contained the highest concentrations of glucose and fructose, they exhibited the lowest browning index among the dried samples, suggesting that the considerably shorter drying time limited cumulative thermal exposure and consequently reduced extensive non-enzymatic browning.

Overall, the results demonstrated that fructose and glucose are the dominant sugars in dried jujube fruits, consistent with previous reports [1,12,31], and that drying temperature significantly influences the balance between sucrose and reducing sugars. Among the tested treatments, drying at 50 °C was more effective in preserving sucrose, whereas drying at 70 °C enhanced glucose and fructose concentrations.

#### 2.3.2. Effects of Drying Temperature on Organic Acid Composition

Organic acids are among the major determinants of jujube fruit flavor quality. Therefore, the significant changes observed in malic, citric, succinic and oxalic acids following drying may directly influence the sensory characteristics of dried jujube products [34]. The organic acid composition of jujube fruits was significantly influenced by drying temperature (*p* ≤ 0.01) (Table 2). In general, all organic acids were considerably higher in dried samples than in fresh fruits, reflecting the concentration effect associated with moisture loss during drying.

Malic acid was the major organic acid in all treatments, ranging from 494.25 mg 100 g^−1^ in fresh fruits to 2002.43 mg 100 g^−1^ in fruits dried at 50 °C. Similar findings have been reported in previous studies, where malic acid was identified as the major organic acid in jujube fruits [31,34,35]. The highest malic acid concentration was observed at 50 °C, followed by 60 °C and 70 °C, showing its decrease with increasing temperature. Malic acid, a major intermediate of the tricarboxylic acid (TCA) cycle, plays a crucial role in plant carbon metabolism. In addition to its role in fruit acidity, malate serves as a vital carbon and reducing equivalent store during fruit development and post-harvest metabolism [36]. The gradual decrease in malic acid content with increasing drying temperature indicates lower thermal stability of malate in prolonged hot-air drying. In addition to the concentration effect due to water loss, differences in the thermal stability of individual organic acids may also contribute to the observed changes in their concentrations [37].

However, in a study, oxalic acid was identified as the predominant organic acid, which contrasts with the findings of the present study as well as those of other previously published studies [17]. The biochemical composition, including organic acids, of jujube is inherently variable and influenced by multiple factors, including genotype, ecotype, environmental conditions, and harvest maturity stage [11,12,14,38]. Citric acid was the second most abundant organic acid, with significantly higher levels in dried fruits (657.73–675.62 mg 100 g^−1^) than in fresh fruits (183.53 mg 100 g^−1^). Similar trends have been reported by [31,35], where citric acid was identified as one of the major acids contributing to fruit acidity. Citric acid is an important intermediate in the tricarboxylic acid (TCA) cycle and plays a significant role in cellular energy metabolism as well as in determining fruit acidity and aroma [36]. Although citric acid concentration increased significantly after drying, no significant difference was observed between drying temperatures. This finding suggests that citric acid is relatively stable under the applied drying conditions and that the increase in its concentration is primarily related to water removal rather than extensive thermal degradation. Therefore, citric acid retention may help maintain the acidity and aroma balance of dried jujube fruit. Tong et al. [34] reported that malic and citric acids gradually accumulated during fruit development and became the dominant organic acids at fruit maturity. The high concentrations of these acids observed in dried fruits in the present study further emphasize their importance as major factors affecting the quality of jujube fruit. Tong et al. [34] demonstrated that malic and citric acid metabolism are closely interconnected and regulated by common metabolic pathways in jujube fruits. Similarly, Wei et al. [39] suggested that organic acid accumulation depends on the balance between synthesis, degradation, transport, and storage processes. Therefore, the differences observed among drying temperatures in the present study may not be solely attributed to moisture loss but also to the differential stability of individual organic acids during thermal processing.

Increasing drying temperature led to an increase in the content of succinic acid, and its value was the highest at 70 °C (623.76 mg 100 g^−1^), whereas the lowest concentration was found in fresh fruits (97.67 mg 100 g^−1^). A similar trend was observed for fumaric acid, which increased from 1.23 mg 100 g^−1^ in fresh fruits to 18.70 mg 100 g^−1^ at 70 °C. Oxalic acid concentrations were also significantly raised by drying, with the highest level detected at 70 °C (95.65 mg 100 g^−1^). The gradual decrease in malic acid content with increasing drying temperature suggests that high temperatures may accelerate malate degradation or reduce its stability during processing [39]. Citric acid metabolism is also closely linked to the tricarboxylic acid cycle, and its accumulation depends on the balance between synthesis and degradation pathways. The relatively stable citric acid concentrations observed at different drying temperatures suggest that citric acid may be more resistant to thermal degradation than malic acid under the conditions applied in this study [39].

Shikimic acid, on the other hand, showed a different response to drying temperature. The highest shikimic acid concentration was recorded in fruits dried at 50 °C (18.78 mg 100 g^−1^), while the lowest value was observed at 60 °C (3.13 mg 100 g^−1^). Shikimic acid is an important intermediate in the shikimate pathway, providing precursors for the biosynthesis of aromatic amino acids and numerous phenolic compounds via the phenylpropanoid pathway [40]. The decline in shikimic acid at higher drying temperatures suggests that this metabolite may be susceptible to heat-induced degradation during the drying process. Fresh fruits contained intermediate levels of shikimic acid (9.61 mg 100 g^−1^). The higher shikimic acid concentration observed at 50 °C suggests that moderate drying temperatures were more effective in preserving this metabolite than higher drying temperatures and may also be associated with improved release or extractability of shikimic acid from the fruit matrix under moderate drying conditions. The particularly low concentration observed at 60 °C may reflect the combined effects of drying temperature and exposure time rather than temperature alone. Although the temperature was lower than 70 °C, drying at 60 °C required a longer period, which may have increased cumulative thermal and oxidative degradation. In contrast, the shorter drying duration at 70 °C may have limited prolonged exposure, resulting in a slightly higher residual shikimic acid concentration [41].

In general, the concentrations of organic acids in jujube fruits were significantly increased by drying in comparison with fresh samples. Lower drying temperatures (50 °C) led to the preservation of malic and shikimic acids, while higher temperatures (70 °C) stimulated higher accumulation of succinic, fumaric, and oxalic acids.

#### 2.3.3. Effects of Drying Temperature on Total Phenolic Content and Antioxidant Activity

The total phenolic content and antioxidant activity of jujube fruits were significantly affected by drying temperature (*p* ≤ 0.01) (Table 3). Drying resulted in a significant increase in total phenolic content compared to fresh fruit; however, the degree of this increase varied depending on the drying temperature applied. The highest total phenolic content was observed in fruit dried at 50 °C (5393.43 mg GAE/100 g), followed by samples dried at 60 °C (3399.06 mg GAE/100 g) and 70 °C (2402.10 mg GAE/100 g). Fresh fruit showed the lowest phenolic content (414.93 mg GAE/100 g). The significant increase in phenolic concentration after drying can be attributed to moisture loss and the concentration of bioactive compounds. However, the gradual decrease in total phenolic content with increasing drying temperature suggests that high temperatures may increase the degradation of heat-sensitive phenolic compounds.

The total phenolic contents obtained in this study were generally comparable to or higher than many values reported in the literature. Gao et al. [8] reported total phenolic contents ranging from 600.4 to 4289.5 mg kg^−1^ FW, whereas Ghani et al. [6] reported values between 16.11 and 32.90 mg GAE g^−1^. Similarly, studies conducted on Chinese [42,43], Tunisian [44,45], and Spanish jujube fruits [13,46] reported phenolic contents ranging from 0.557 to 21.61 mg GAE g^−1^ depending on genotype, geographical origin, and maturity stage. The fresh fruit phenolic content observed in the present study was also within the range reported by Sagbas [1], who found total phenolic contents between 293.19 and 992.48 mg GAE 100 g^−1^ FW in different jujube ecotypes. Similarly, Colak and Alan [17] reported a total phenolic content of 379.33 mg GAE L^−1^, which is comparable to the results of fresh fruit value in this study. In contrast, Yan et al. [12] reported considerably higher phenolic concentrations in lyophilized samples, ranging from 5.92 to 35.06 g GAE kg^−1^ DW, highlighting the strong influence of dehydration method on phenolic retention.

A similar trend was observed for antioxidant activity measured via DPPH radical scavenging capacity. The highest DPPH inhibition value was recorded in fruits dried at 50 °C (73.56%), indicating superior antioxidant potential. Fresh fruits showed moderate antioxidant activity (59.75%), while the lowest values were detected in samples dried at 60 °C (54.76%) and 70 °C (54.05%), which did not differ significantly from each other. The higher DPPH activity observed in fruits dried at 50 °C compared with fresh fruits may be attributed to enhanced extractability of phenolic antioxidants during moderate drying, which promotes cell-wall loosening and improves solvent accessibility [47,48]. This interpretation is also supported by the higher total phenolic content observed at 50 °C in the present study. The highest DPPH value obtained in the present study was similar to the lower range reported by Ghani et al. (75–90%), although slightly lower than the values reported by [17] (89.67%) and [1] (79.72–84.89%). These differences may be associated with genotype, fruit maturity, environmental conditions, and extraction procedures. Nevertheless, the relatively high antioxidant activity observed in the present study confirms the strong antioxidant potential of jujube fruits.

Previous drying studies have reported conflicting results regarding the effects of heat treatment on phenolic compounds and antioxidant activity. Tepe et al. [18] observed a significant decrease in total phenolic content from 1911.40 to 418.59–484.03 mg GAE 100 g^−1^ dry weight after drying, accompanied by a 59–61% decrease in antioxidant capacity. Similarly, Anjum et al. [3] reported that the total phenolic content in dried jujube fruit ranged from 137.97 to 243.04 μg GAE mL^−1^ and antioxidant activities ranged from 16.83% to 40.58%. In contrast, the present study showed a remarkable increase in total phenolic content, particularly after drying at 50 °C. Conflicting findings reported in the literature indicate that the response of phenolic compounds to drying is largely dependent on variety characteristics, drying conditions, and extraction protocols. Overall, the present findings indicate that drying at 50 °C provided the best balance between moisture removal and preservation of bioactive compounds.

The increase in total phenolic content after drying cannot be explained solely by the concentration effect resulting from water loss. While moderate heating can enhance the extraction of phenolic compounds by disrupting cell walls and weakening their interactions with structural polysaccharides, partial inactivation of oxidative enzymes such as polyphenol oxidase (PPO) and peroxidase (POD) can further reduce enzymatic oxidation, thus increasing the recovery of extractable phenolics [49,50]. The highest total phenolic content observed at 50 °C indicates that moderate drying establishes a balance between increased phenolic release and limited thermal degradation, while higher temperatures increase the oxidation and polymerization of heat-sensitive phenolics, reducing their recovery via the Folin–Ciocalteau method. Shikimic acid, being an important intermediate in the shikimate pathway that provides precursors for the biosynthesis of aromatic amino acids and phenolic compounds, demonstrates that its higher stability at 50 °C and high total phenolic content indicate that moderate drying conditions are more effective in preserving metabolites belonging to this pathway [40]. It should also be noted that the Folin-Ciocalteu test measures the total reducing capacity of the extract, not individual phenolic compounds. However, the close correlation between total phenolic content and DPPH radical scavenging activity suggests that phenolic compounds are the components that contribute most significantly to the antioxidant capacity of dried jujube fruits. The antioxidant activity of phenols is primarily related to their ability to donate hydrogen atoms or electrons via hydroxyl groups attached to aromatic rings; differences in hydroxylation pattern and molecular structure affect radical scavenging efficiency [51,52].

#### 2.3.4. Principal Component Analysis (PCA) and Heatmap Evaluation

**Figure 2:**
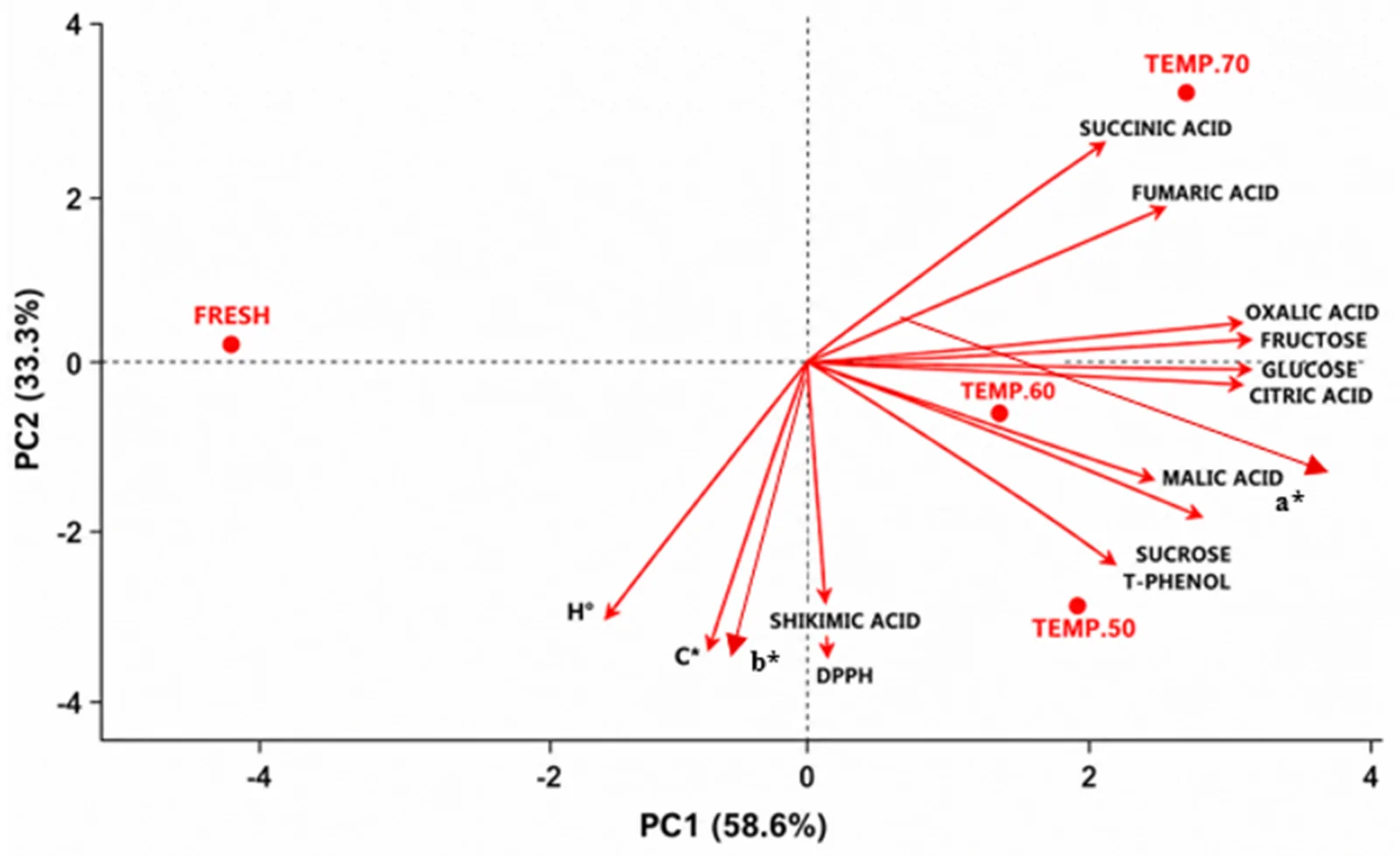
Principal component analysis (PCA) biplot showing the relationships among drying treatments and quality attributes of fresh and dried jujube fruits.

Principal component analysis (PCA) was performed to evaluate the relationships between biochemical parameters and drying processes of jujube fruits (Figure 2). The first two principal components explained 91.9% of the total variation; PC1 and PC2 accounted for 58.6% and 33.3%, respectively. Fresh and dried samples were clearly separated along PC1, indicating significant biochemical changes caused by the drying process.

The PCA biplot showed a clear relationship between drying processes and quality characteristics. The 50 °C process was closely associated with total phenolic content, DPPH radical scavenging activity, sucrose, malic acid, and shikimic acid, suggesting that the medium drying temperature helps preserve bioactive compounds and important quality-related metabolites. In contrast, the 70 °C process was strongly associated with succinic acid, fumaric acid, and oxalic acid, indicating that higher drying temperatures cause changes in organic acid composition. The 60 °C treatment exhibited transitional biochemical characteristics, occupying an intermediate position between the 50 °C and 70 °C treatments. Fresh fruits were clearly separated from dried samples and showed a closer correlation with color parameters, particularly L* and h° values.

**Figure 3:**
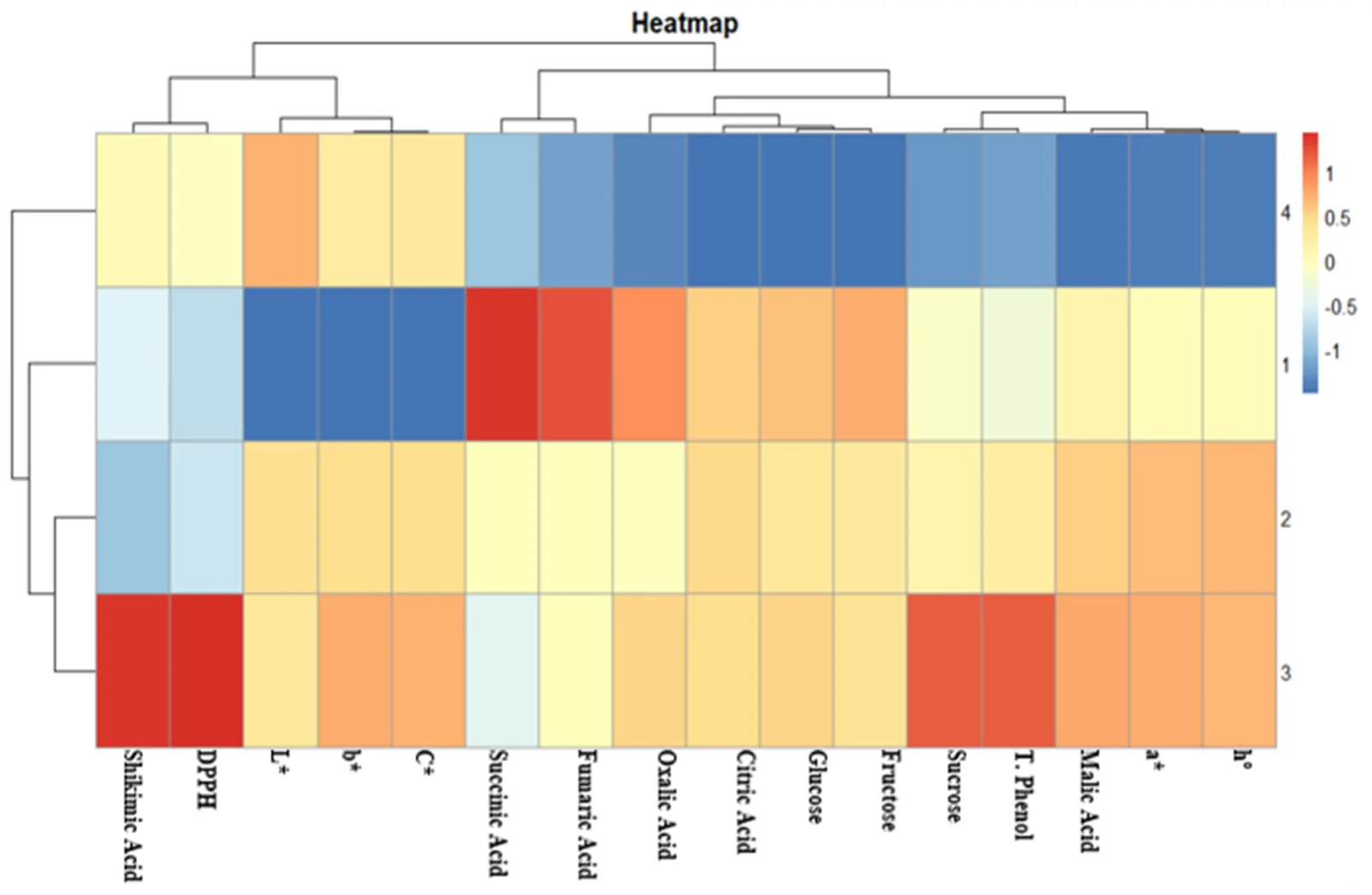
Heatmap and hierarchical cluster analysis of biochemical and quality parameters in fresh and dried jujube fruits subjected to different drying temperatures. Numbers on the *y*-axis represent treatment groups as follows: 1 = 70 °C, 2 = 60 °C, 3 = 50 °C, and 4 = fresh fruits.

Heatmap analysis also validated the PCA results by clustering both drying processes and biochemical properties according to similarity patterns (Figure 3). Two main sample groups were observed. Fresh fruits formed a distinct cluster characterized by higher color parameter values and lower concentrations of sugar, organic acids, phenolic compounds, and antioxidant activity. In contrast, dried samples formed a separate cluster showing significant compositional changes after drying.

Among the dried processes, 50 °C samples were characterized by higher levels of shikimic acid, DPPH activity, total phenolic compound content, sucrose, and malic acid, while the 70 °C process showed higher concentrations of succinic acid, fumaric acid, and oxalic acid. The 60 °C process generally showed intermediate values for most variables. The clustering pattern confirmed that drying temperature significantly affected the biochemical profile of jujube fruits, with 50 °C being more effective in preserving bioactive compounds and 70 °C being associated with greater changes in organic acid composition. It showed a stronger correlation with color parameters, especially L* and h° values.

Drying at 70 °C greatly shortened the drying time and improved the preservation of color, but biochemical analysis showed that 50 °C was more effective in maintaining bioactive compounds and antioxidant capacity. These results were supported by PCA, which showed a close association between 50 °C treatment and total phenolic content, DPPH activity, sucrose, malic acid and shikimic acid. Therefore, the optimal drying temperature can vary depending on the final application of the product. However, while 70 °C is preferred for industrial processing efficiency and color retention, 50 °C appears to be more suitable when the primary objective is to preserve nutritional and functional quality attributes.

### 2.4. Classical Model Results

**Figure 4:**
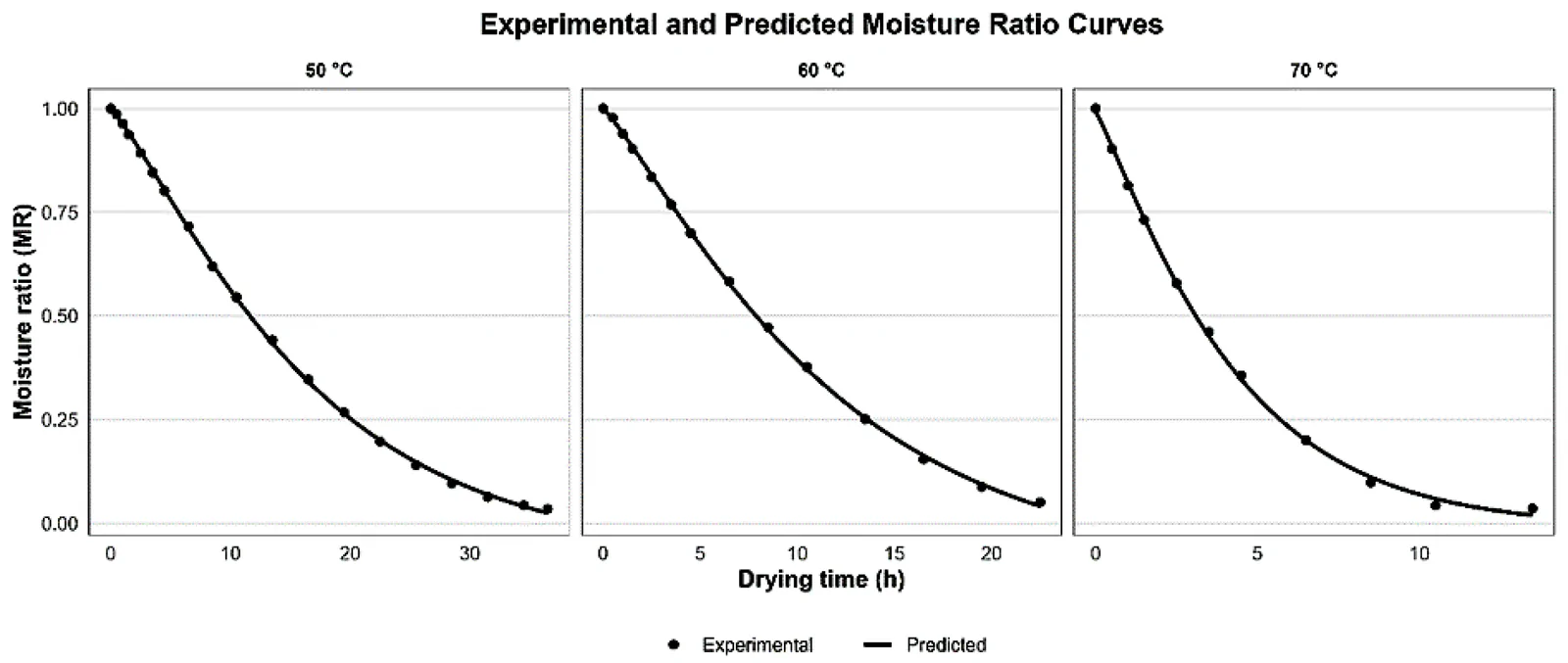
Experimental and Predicted Drying Curves of Jujube Fruits at Different Drying Temperatures.

The comparison of classical thin-layer drying models on training data (H1–H3) is given in Table 4. The best performance was obtained with the Midlli-Kucuk model at all drying temperatures. Therefore, the Midlli-Kucuk model was selected in the next stage, and the agreement between experimental data and model predictions is shown in Figure 4. The close agreement between experimental and predicted values confirmed that the Midlli-Kucuk model adequately described the drying behavior of jujube fruits.

The parameters of the selected best classical model and the H4 validation results are given in Table 5. On the independent H4 validation dataset, the Midlli-Kucuk model maintained the best fit at 50 and 70 °C, while at 60 °C, alternative models showed similar performance with very small differences. Overall, the Midlli-Kucuk model stands out as the most stable classical model across the temperature range.

### 2.5. Artificial Neural Network Results

**Figure 5:**
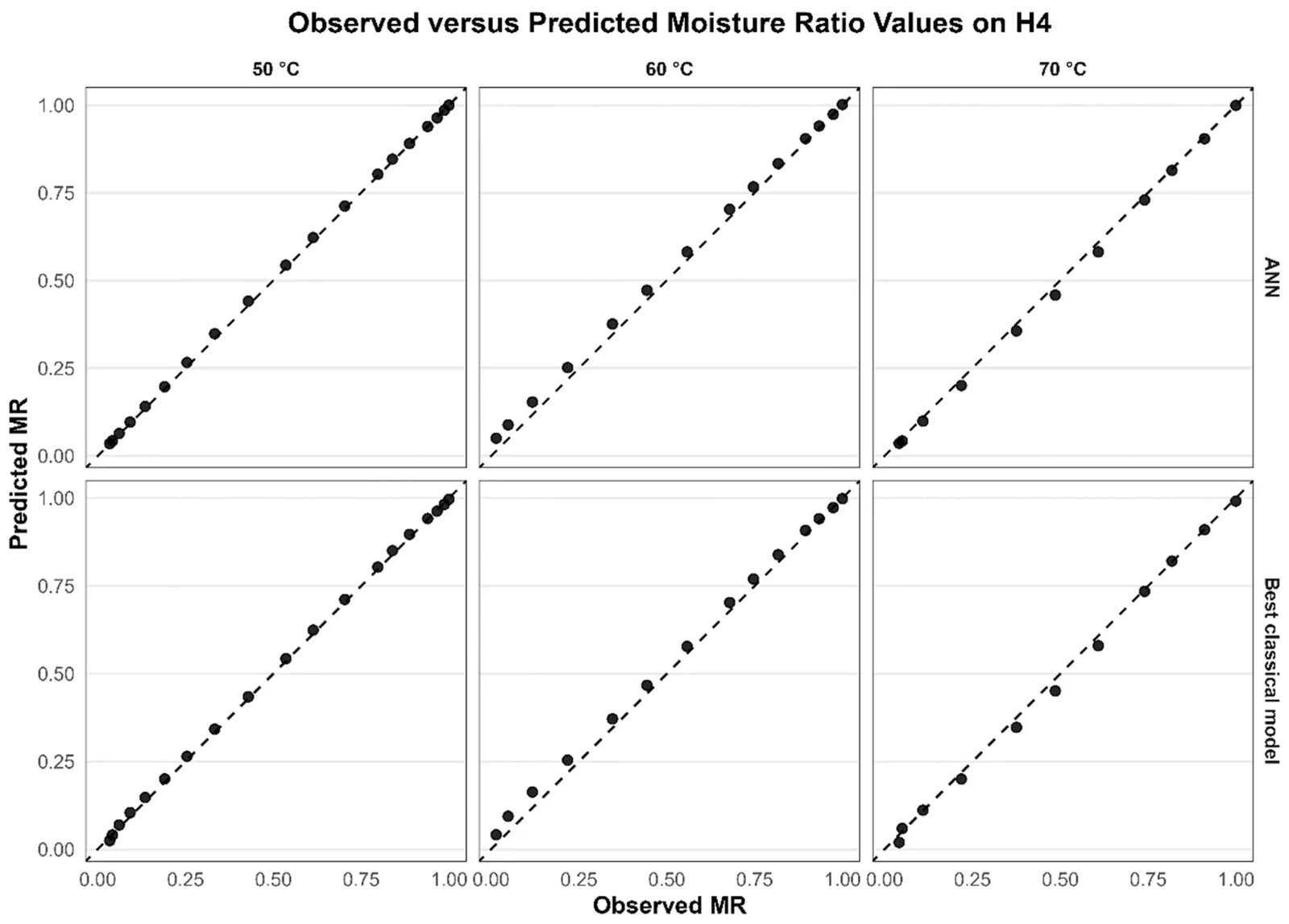
Observed versus Predicted Moisture Ratio (MR) Values for the ANN and Best Classical Model on the Independent H4 Validation Dataset.

The best performance in artificial neural network analyses was obtained with the 39-neuron model. The H4 validation results of the ANN model showed that it yielded lower error values at all temperatures compared to the classical model (Table 6). The relationship between the observed and predicted values is presented in Figure 5, and it was determined that the ANN model showed high fit to the experimental data.

### 2.6. Comparison of ANN with the Best Classical Model

**Figure 6:**
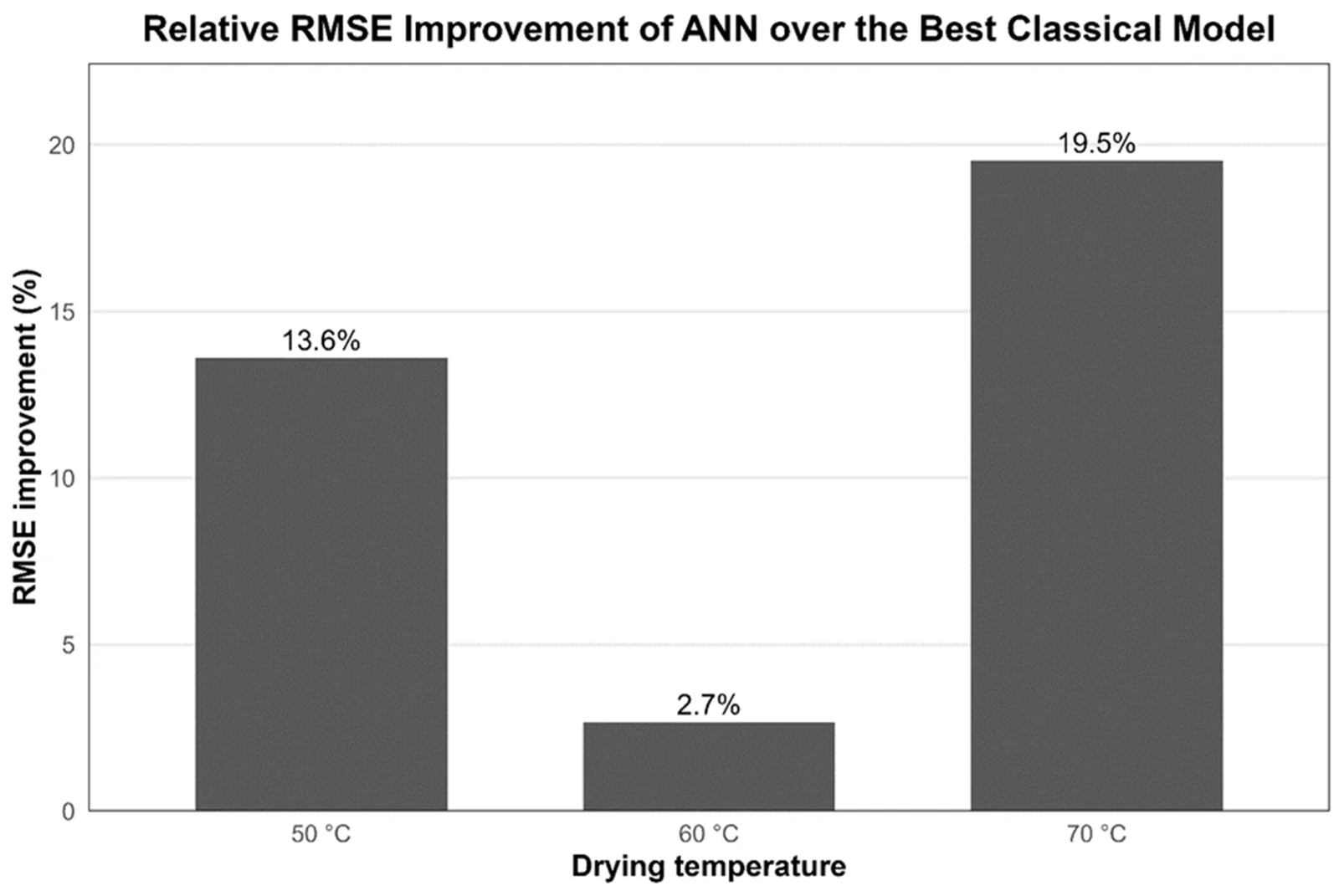
Relative RMSE Improvement (%) of the ANN Model over the Best Classical Model at Different Drying Temperatures.

Based on the H4 validation dataset, compared with the best classical model, the ANN approach performed better than the classical model at all temperatures (Table 6). The RMSE improvement achieved by the ANN model was 13.60% at 50 °C, 2.67% at 60 °C, and 19.52% at 70 °C. In the overall H4 dataset, the total RMSE improvement of the ANN model compared to the classical model was 7.76%. The temperature-dependent RMSE improvement rates are given in Figure 6.

The results showed that the drying time of jujube fruit decreased with increasing temperature during hot-air drying. Among the classical thin-layer drying models, the Midilli–Kucuk model emerged as the most suitable model overall. This result is consistent with the literature reporting that this model shows strong performance in explaining the drying kinetics of agricultural products [20].

The artificial neural network model gave lower error values in the H4 validation data compared to the classical model. This shows that the nonlinear structure of the drying process can be represented more successfully with the ANN. However, it was also seen that classical models offer an explainable and robust structure. Therefore, the ANN approach can be considered as a complement to classical modeling [23].

The improvement provided by the ANN model varied depending on drying temperature. The relatively lower improvement at 60 °C may be related to the high accuracy already achieved by the classical models under this condition. In particular, the Midilli–Kucuk model showed strong predictive performance, leaving limited residual error for further reduction by the ANN. This suggests that the drying behavior at 60 °C may have been more linear or more readily captured by the empirical assumptions of the Midilli–Kucuk model, thereby reducing the additional predictive advantage of the ANN model. Similar observations have been reported in drying studies where highly accurate empirical models reduced the additional predictive gain obtained from artificial neural networks. Therefore, the advantage of the ANN model appears to be temperature-dependent rather than uniform across all drying conditions [22].

In general, the Midilli-Kucuk model stands out as a strong classical option in jujube drying, while the artificial neural network is an approach that provides higher prediction accuracy. When color findings are evaluated together, the 70 °C application appears to be a remarkable option in terms of drying time and color preservation.

## 3. Materials and Methods

Jujube (*Ziziphus jujuba* Mill.) fruits were harvested from a commercial jujube orchard located in Amasya province, Türkiye (4512896.06′ N latitude and 732665.50′ E longitude), where trees were managed under standard horticultural practices. The orchard was maintained following conventional cultivation techniques, including regular irrigation, fertilization, and pest management. Fruits were collected at the commercial maturity stage based on external color and firmness criteria.

Fresh jujube fruits were selected based on uniformity in size, shape, color, and the absence of visible defects or mechanical damage. The fruits were thoroughly washed with distilled water to remove surface impurities and allowed to air-dry at room temperature before sample preparation. Immediately after harvest, the samples were transported to the laboratory and divided into two groups: fresh and dried samples. Fresh samples used in the experiments were cut in half and their seeds removed. A portion of the fruits was used directly for biochemical analyses in the fresh state. The remaining fruits were subjected to drying treatments under controlled laboratory conditions. An average of 45.0 ± 1.0 g of sample was used to calculate the initial moisture content on a wet basis before drying. Moisture determination was performed in an oven at 70 °C, drying in four parallel rows until the weight changes were constant [53]. Moisture content on both wet and dry bases was calculated using the following equation:

MC_db_ = [(WI − WF)/WF] × 100

Here;

MC_db_: Moisture content according to dry base (g moisture/g dry matter)

WI: Initial weight (g)

WF: Constant weight or final weight after drying (g)

### 3.1. Drying Application

Drying was performed using hot air at three different temperature levels: 50 °C, 60 °C, and 70 °C. Hot-air drying is a widely preferred method in fruits and vegetables due to both its ease of application and the ability to model drying kinetics effectively [18,20]. The fruits were dried until a constant weight was achieved to ensure uniform moisture reduction across treatments. After drying, samples were cooled to room temperature and stored in airtight containers until further analysis. Moisture ratio (MR) was used in the evaluation of drying data. In thin-layer drying studies, modeling MR values as a function of time is a standard approach to compare the effect of different temperatures on drying behavior [20,22]. All samples, including fresh and dried groups, were used for subsequent biochemical analyses to evaluate the effects of drying temperature on the chemical composition and functional properties of jujube fruits.

### 3.2. Color Determination

Color changes in fresh and dried jujube samples were evaluated using the CIE Lab* color system. In this context, the basic color parameters L*, a*, and b* were determined, and from these values, chroma (C*), hue angle (h°), total color difference (ΔE), and browning index (BI) were calculated. The CIE Lab* system is a widely used standard approach for objectively determining color changes in fresh and processed foods [25,54]. In evaluating color changes occurring during drying processes, parameters such as ΔE and BI are also considered important quality indicators [26].

### 3.3. Extraction and Determination of Sugars and Organic Acids

#### 3.3.1. Extraction Procedure

Extraction of sugars and organic acids was performed using double-deionized water. One gram of dried samples was finely ground and mixed with 25 mL of double-deionized water. For fresh samples, 5 g of homogenized fruit was extracted with 25 mL of double-deionized water. The mixtures were shaken at room temperature for 30 min to ensure efficient extraction of soluble compounds.

Following extraction, the samples were centrifuged at 4000 rpm for 15 min at 4 °C. The supernatants were then filtered and transferred into HPLC vials for subsequent analysis of sugars and organic acids.

#### 3.3.2. Sugar Analysis

Sugars were analyzed using high-performance liquid chromatography (HPLC) (Shimadzu Prominence-i LC-2030C 3D, Kyoto, Japan). Separation was carried out using a Phenomenex Rezex RCM-Monosaccharide column (300 × 7.8 mm). The mobile phase consisted of bidistilled water at a flow rate of 0.6 mL min^−1^. Detection was performed using a refractive index detector (RID) (Shimadzu RID-20A, Kyoto, Japan). Identification and quantification of sugar peaks were achieved by comparison with external standards, including sucrose, glucose, and fructose.

#### 3.3.3. Organic Acid Analysis

Organic acids were determined using the same HPLC system (Shimadzu Prominence-i LC-2030C 3D, Japan). Separation was performed using a Hi-Plex H column (300 × 7.7 mm). The mobile phase consisted of bidistilled water acidified with 0.02 N H_2_SO_4_, delivered at a flow rate of 0.6 mL min^−1^. Detection of organic acids was performed using a UV detector at 210 nm. Organic acid peaks were identified and quantified based on their retention times and comparison with appropriate standards.

### 3.4. Extraction Procedure for Phenolic Compounds and Antioxidant Activity

One gram of finely ground samples was mixed with 10 mL of 80% methanol. The mixture was incubated in an ultrasonic water bath for 2 h at room temperature. Following extraction, all samples were centrifuged at 4000 rpm for 15 min at room temperature. The supernatants were collected and used for subsequent biochemical analyses.

#### 3.4.1. Determination of Total Phenolic Content

Total phenolic content was determined using the Folin–Ciocalteu method as described by [55] with some modifications. Briefly, 300 µL of the extract was mixed with 1500 µL of 0.2 N Folin–Ciocalteu reagent and then 1200 µL of 7.5% sodium carbonate (Na_2_CO_3_) was added. The reaction mixture was incubated at room temperature for 2 h in the dark. After incubation, the absorbance was measured at 760 nm using a spectrophotometer. Total phenolic content was calculated based on a gallic acid calibration curve and expressed as mg gallic acid equivalents (GAE) per 100 g dry weight in dried samples and as fresh weight in fresh samples.

#### 3.4.2. Determination of DPPH Radical Scavenging Activity

The antioxidant activity of the samples was evaluated using the DPPH radical scavenging assay [56]. An aliquot of 0.05 mL of extract was mixed with 0.1 mM DPPH solution and the final volume was adjusted to 2 mL. The reaction mixtures were incubated in the dark for 30 min at room temperature. Following incubation, absorbance was measured at 517 nm using a spectrophotometer. The results were expressed as percentage inhibition of the DPPH radical, calculated using the following equation:

$$\mathrm{Inhibition}\;(\%)=\frac{A_{\mathrm{DPPH}}-A_{\mathrm{sample}}}{A_{\mathrm{DPPH}}}\times 100$$

where $A_{\mathrm{DPPH}}$ is the absorbance of the control and $A_{\mathrm{sample}}$ is the absorbance of the sample.

### 3.5. Statistical Analysis

All analyses were performed in triplicate, and the results were expressed as mean ± standard deviation. The data were subjected to one-way analysis of variance (ANOVA) to evaluate the effects of different drying temperatures on the measured parameters. Mean comparisons were carried out using the Least Significant Difference (LSD) test at significance levels of *p* ≤ 0.05 and *p* ≤ 0.01.

Statistical analyses were conducted using JMP^®^ Pro 18.0.0 software (SAS Institute Inc., Cary, NC, USA). Multivariate analyses were performed using the R version 4.6.1 (R Core Team, Vienna, Austria)environment. PCA was used to identify patterns and relationships among samples and variables, while heatmap analysis was applied to visualize the distribution and clustering of biochemical traits across treatments.

### 3.6. Classical Thin-Layer Drying Models

To explain drying behavior, Lewis, Page, Henderson and Pabis, Logarithmic, Wang and Singh, and Midilli–Kucuk models were evaluated. These models are among the most widely used empirical and quasi-empirical models in describing the drying kinetics of agricultural products [20,22,57]. In comparing model performances, the coefficient of determination (R^2^), root mean square error (RMSE), and reduced chi-square (χ^2^) values were considered. Information criteria were also included in the model comparison when necessary. In this study, modeling was carried out in two stages. H1, H2, and H3 replicates were used for model training, while the H4 replicate was reserved as the independent validation set. Thus, the aim was to identify models that showed not only high fit to the training data but also strong performance in the independent validation dataset.

### 3.7. Artificial Neural Network Model

In addition to classical models, an artificial neural network (ANN) approach was used for estimating drying data. The literature also reports that ANN models are successfully used in predicting drying kinetics and are considered as an alternative or complementary method to classical thin-layer models [23,58,59,60,61]. In this study, the ANN model was constructed in a multilayer feedforward network structure that takes drying time and temperature as input and moisture content (MR) as output. The variables were normalized to the 0–1 range, the optimal number of hidden neurons was selected by performing iteration-based validation mappings and repeated cross-validation, and the final model was tested on the independent H4 dataset.

## 4. Conclusions

This study investigated the effects of hot-air drying at different temperatures on the drying behavior, color characteristics, sugar and organic acid composition, and bioactive compound content of jujube fruits. Increasing the drying temperature significantly reduced the drying time, such that the application of 70 °C provided the shortest drying time. Among the classical thin-film drying models, the Midilli-Kucuk model was determined as the most successful model at all temperatures, while the artificial neural network (ANN) model showed higher prediction accuracy and proved to be a strong alternative in modeling the drying kinetics. The drying process significantly changed the biochemical composition of jujube fruits. The sugar and organic acid concentrations in the dried samples increased significantly compared to the fresh fruits. The application of 50 °C was more effective in preserving sucrose, malic acid, and shikimic acid, while the application of 70 °C resulted in increased levels of glucose, fructose, succinic acid and fumaric acid. The highest values for total phenolic content and antioxidant activity were obtained at 50 °C and a gradual decrease in these parameters was observed with increasing temperature. In contrast, when the color characteristics were evaluated, the 70 °C treatment provided better color retention with lower values of total color change and browning index. PCA and heatmap analyses confirmed that drying temperature significantly affected the biochemical characteristics of jujube fruits. The 50 °C treatment was associated with total phenolic content, DPPH activity, sucrose, and malic acid, while the 70 °C treatment was associated with succinic, fumaric, and oxalic acids. Overall, 50 °C treatment appeared as the most suitable temperature for preserving bioactive compounds and antioxidant properties, while 70 °C treatment provided shorter drying time and better color retention. These results indicate that the choice of temperature to determine the optimal drying conditions in jujube fruit processing should be based on the desired quality characteristics of the product. From a practical point of view, drying at 50 °C is recommended when the main objective is to maximize the retention of bioactive compounds and antioxidant properties, while 70 °C may be chosen for industrial applications where shorter processing time and better color preservation are needed. While this study provides valuable insights into the effects of hot-air drying on jujube fruit quality, only a limited range of drying temperatures and a single drying method were evaluated. Future studies comparing different drying techniques and a broader range of processing conditions would further contribute to optimizing product quality and energy efficiency.

## Figures and Tables

**Table 1:** Effects of fresh and different drying temperatures on sugar composition of jujube fruit (%).

Treatments	Sucrose	Glucose	Fructose
**Temp.70**	17.76 ± 0.90 ^c^	24.26 ± 0.51 ^a^	24.62 ± 0.34 ^a^
**Temp.60**	19.96 ± 0.67 ^b^	21.55 ± 0.42 ^b^	20.72 ± 1.77 ^b^
**Temp.50**	29.32 ± 0.95 ^a^	23.05 ± 1.91 ^ab^	21.35 ± 1.06 ^b^
**Fresh**	7.68 ± 0.09 ^d^	4.72 ± 0.09 ^c^	4.61 ± 0.01 ^c^
**LSD_%1_**	1.381	1.903	1.967

Values are expressed as mean ± standard deviation (n = 3). Different lowercase letters within the same column indicate significant differences among treatments according to the LSD test at *p* ≤ 0.01. Temp.: Temperature, LSD: Least Significant Difference.

**Table 2:** Effects of fresh and different drying temperatures on organic acid composition of jujube fruit (mg100 g^−1^).

Treatments	Malic Acid	Citric Acid	Succinic Acid	Oxalic Acid	Fumaric Acid	Shikimic Acid
**Temp.70**	1558.73 ± 75.46 ^c^	675.62 ± 4.88 ^a^	623.76 ± 5.18 ^a^	95.65 ± 2.14 ^a^	18.7 ± 0.52 ^a^	5.91 ± 0.03 ^c^
**Temp.60**	1854.86 ± 15.18 ^b^	665.60 ± 3.06 ^a^	302.63 ± 7.27 ^b^	64.88 ± 0.87 ^c^	9.73 ± 0.31 ^b^	3.13 ± 0.03 ^d^
**Temp.50**	2002.43 ± 32.99 ^a^	657.73 ± 18.74 ^a^	200.15 ± 6.03 ^c^	83.27 ± 1.00 ^b^	9.62 ± 0.33 ^b^	18.78 ± 0.51 ^a^
**Fresh**	494.25 ± 6.39 ^d^	183.53 ± 2.47 ^b^	97.67 ± 0.47 ^d^	22.86 ± 0.90 ^d^	1.23 ± 0.02 ^c^	9.61 ± 0.03 ^b^
**LSD_%1_**	79.079	18.639	10.139	2.519	0.65	0.484

Values are expressed as mean ± standard deviation (n = 3). Different lowercase letters within the same column indicate significant differences among treatments according to the LSD test at *p* ≤ 0.01. Temp.: Temperature, LSD: Least Significant Difference.

**Table 3:** Effects of fresh and different drying temperatures on total phenolic content and DPPH radical scavenging activity of jujube fruit.

Treatments	Total Phenolic (mg GAE 100 g^−1^)	DPPH (%)
**Temp.70**	2402.1 ± 67.68 ^c^	54.05 ± 1.67 ^c^
**Temp.60**	3399.06 ± 67.03 ^b^	54.76 ± 1.30 ^c^
**Temp.50**	5393.43 ± 74.12 ^a^	73.56 ± 1.27 ^a^
**Fresh**	414.93 ± 13.8 ^d^	59.75 ± 0.31 ^b^
**LSD_%1_**	114.364	2.034

Values are expressed as mean ± standard deviation (n = 3). Different lowercase letters within the same column indicate significant differences among treatments according to the LSD test at *p* ≤ 0.01. Temp.: Temperature, LSD: Least Significant Difference.

**Table 4:** Comparison of Classical Drying Models Based on Training Data.

Temperature (°C)	Model	RMSE	R^2^	χ^2^	Rank
50	Midilli-Kucuk	0.00498	0.9998	0.000031	1
50	Wang and Singh	0.00773	0.99952	0.000067	2
50	Page	0.01077	0.99908	0.00013	3
50	Logarithmic	0.01314	0.99863	0.000205	4
50	Henderson and Pabis	0.03548	0.98999	0.001407	5
50	Lewis	0.04361	0.98488	0.002008	6
60	Midilli-Kucuk	0.00509	0.99977	0.000036	1
60	Wang and Singh	0.00762	0.99947	0.000068	2
60	Logarithmic	0.01118	0.99887	0.000159	3
60	Page	0.0116	0.99878	0.000157	4
60	Henderson and Pabis	0.03466	0.98914	0.001401	5
60	Lewis	0.04255	0.98362	0.00195	6
70	Midilli-Kucuk	0.00984	0.99916	0.000152	1
70	Page	0.01058	0.99902	0.000137	2
70	Logarithmic	0.01428	0.99822	0.00028	3
70	Wang and Singh	0.01961	0.99664	0.00047	4
70	Henderson and Pabis	0.02057	0.99631	0.000517	5
70	Lewis	0.02337	0.99524	0.000601	6

**Table 5:** Parameters of the Optimal Classical Model and H4 Validation Results.

Temp. (°C)	Selected Model	A	k	n	B	RMSE (H4)	R^2^ (H4)	χ^2^ (H4)
50	Midilli-Kucuk	0.99657	0.03357	1.21477	−0.00124	0.00816	0.99948	0.000084
60	Midilli-Kucuk	0.99802	0.05577	1.188696	−0.00280	0.0261	0.99427	0.000954
70	Midilli-Kucuk	0.99124	0.18854	1.137619	−0.00047	0.01878	0.99689	0.000554

**Table 6:** Performance of the Artificial Neural Network Model on the Independent H4 Validation Dataset.

Comparison Level	RMSE (ANN)	R^2^ (ANN)	RMSE Improvement (%)
50 °C	0.00705	0.99961	13.6
60 °C	0.02541	0.99457	2.67
70 °C	0.01512	0.99798	19.52
Overall H4	0.01685	0.99766	7.76

## Data Availability

The original contributions presented in this study are included in the article. Further inquiries can be directed to the corresponding author.

## Abbreviations

The following abbreviations are used in this manuscript:

ANOVA	Analysis of Variance
DPPH	2,2-Diphenyl-1-picrylhydrazyl
GAE	Gallic Acid Equivalents
HPLC	High-Performance Liquid Chromatography
LSD	Least Significant Difference
PCA	Principal Component Analysis
RID	Refractive Index Detector
SDG	Sustainable Development Goal
TCA	Tricarboxylic Acid
